# Spatial Heterogeneity in Large Resected Diffuse Large B-Cell Lymphoma Bulks Analysed by Massively Parallel Sequencing of Multiple Synchronous Biopsies

**DOI:** 10.3390/cancers13040650

**Published:** 2021-02-06

**Authors:** Teresa Magnes, Sandro Wagner, Aaron R. Thorner, Daniel Neureiter, Eckhard Klieser, Gabriel Rinnerthaler, Lukas Weiss, Florian Huemer, Konstantin Schlick, Nadja Zaborsky, Markus Steiner, Richard Greil, Alexander Egle, Thomas Melchardt

**Affiliations:** 1 Department of Internal Medicine III with Haematology, Medical Oncology, Haemostaseology, Infectiology and Rheumatology, Oncologic Center, Paracelsus Medical University, Müllner Hauptstraße 48, 5020 Salzburg, Austria; t.magnes@salk.at (T.M.); sa.wagner@salk.at (S.W.); g.rinnerthaler@salk.at (G.R.); lu.weiss@salk.at (L.W.); f.huemer@salk.at (F.H.); k.schlick@salk.at (K.S.); r.greil@salk.at (R.G.); a.egle@salk.at (A.E.); n.zaborsky@salk.at (N.Z.); mark.steiner@salk.at (M.S.); 2 Center for Cancer Genomics, Dana-Farber Cancer Institute, 450 Brookline Ave, Boston, MA 02215, USA; Aaron_Thorner@dfci.harvard.edu; 3 Department of Pathology, Paracelsus Medical University, Müllner Hauptstraße 48, 5020 Salzburg, Austria; d.neureiter@salk.at (D.N.); e.klieser@salk.at (E.K.); 4 Cancer Cluster Salzburg, Müllner Hauptstraße 48, 5020 Salzburg, Austria; 5 Salzburg Cancer Research Institute-Laboratory for Immunological and Molecular Cancer Research (SCRI-LIMCR), Müllner Hauptstraße 48, 5020 Salzburg, Austria

**Keywords:** diffuse large B-cell lymphoma, massively parallel sequencing, spatial heterogeneity, clonal evolution, lymphomagenesis

## Abstract

**Simple Summary:**

Substantial genetic heterogeneity was described within large tumour masses of several cancer entities. However, this topic has not been addressed in patients with diffuse large B-cell lymphoma (DLBCL). Therefore, we collected multiple biopsies of twelve patients who had diagnostic or therapeutic resections of large lymphoma bulks and analysed 213 genes known to be important for lymphoma biology. The biopsies of each patient were compared to investigate the spatial heterogeneity in DLBCL. Ten out of twelve patients had discordant mutations which were not present in all of their biopsies and similar results were seen by the analysis of copy number variants. Some of the involved genes have a known prognostic and therapeutic relevance in DLBCL. This shows that single biopsies underestimate the complexity of the disease and might overlook possible mechanisms of resistance and therapeutic targets.

**Abstract:**

Diffuse large B-cell lymphoma (DLBCL) usually needs to be treated immediately after diagnosis from a single lymph node biopsy. However, several reports in other malignancies have shown substantial spatial heterogeneity within large tumours. Therefore, we collected multiple synchronous biopsies of twelve patients that had diagnostic or therapeutic resections of large lymphoma masses and performed next-generation sequencing of 213 genes known to be important for lymphoma biology. Due to the high tumour cell content in the biopsies, we were able to detect several mutations which were present with a stable allelic frequency across all the biopsies of each patient. However, ten out of twelve patients had spatially discordant mutations and similar results were found by the analysis of copy number variants. The median Jaccard similarity coefficient, a measure of the similarity of a sample set was 0.77 (range 0.47–1), and some of the involved genes such as *CARD11*, *CD79B*, *TP53*, and *PTEN* have a known prognostic or therapeutic relevance in DLBCL. This shows that single biopsies underestimate the complexity of the disease and might overlook possible mechanisms of resistance and therapeutic targets. In the future, the broader application of liquid biopsies will have to overcome these obstacles.

## 1. Introduction

Diffuse large B-cell lymphoma (DLBCL) is the most frequent subtype of non-Hodgkin-lymphoma (NHL) and between 3–7 cases per 100,000 persons are diagnosed each year [1,2,3,4]. Even though a cure can be achieved by primary chemoimmunotherapy in more than half of the patients, the prognosis for recurrent or refractory DLBCL is significantly worse [5]. While immunotherapy with genetically engineered T-cells was recently shown to be a promising option for a selected group of these patients, the development of targeted therapies for DLBCL is still lagging behind other tumour entities [6,7]. One reason for the difficulty to establish new drugs for DLBCL linked to the presence of certain molecular targets might be the genetic complexity of the disease. Analyses using next-generation sequencing (NGS) techniques in over 1000 single biopsies of DLBCL patients have revealed more than 150 possible genetic drivers [8] confirming earlier reports on the striking molecular heterogeneity of aggressive NHL [9]. Furthermore, it has been shown in several other entities, such as renal cancer, breast cancer, or lung cancer that there is also a possibility for substantial genetic differences between not only the primary tumour and metastases at the same timepoint but also between multiple sites in one large tumour mass [10,11,12]. By investigating paired samples of DLBCL at the time of primary diagnosis and at relapse of disease, we observed relevant dynamics on the mutational level over time [13]. In order to investigate spatial heterogeneity in DLBCL, we now collected multiple synchronous biopsies of large, resected lymphoma samples and performed NGS of 213 disease related genes.

## 2. Results

### 2.1. Patient Characteristics

Out of a cohort of more than 380 patients diagnosed with DLBCL between 2004 and 2015 at the Third Medical Department of the Paracelsus Medical University, we identified twelve patients who had resections of large lymphoma masses due to diagnostic or therapeutic reasons. In all but one patient (patient 5), surgery took place at the time of primary diagnosis, before any lymphoma-specific treatment was given (see Table 1 for detailed patient characteristics). In patient 5, one cervical lymph node was resected at the time of primary diagnosis and neck dissection and bone marrow biopsy were performed after progression on multiple treatment lines. Two patients (16.7%) had testicular lymphomas, two patients (16.7%) had DLBCL of leg type, and six patients (50.0%) had large abdominal lymphoma masses. According to the Hans classifier, seven patients (58.3%) had the germinal centre B-cell (GCB) such as DLBCL and the *MYC* translocations were found in two (16.7%) patients. Patient 1 had a double-hit lymphoma with a *MYC* translocation and an additional *BCL6* translocation and patient 5 had a triple hit lymphoma with additional *BCL2* and *BCL6* translocations.

We compared the clinical characteristics of the twelve patients who had resections of large lymphoma manifestations to the 331 patients in our database who were treated with at least rituximab and an anthracycline and only had diagnostic biopsies. There were no statistically significant differences in the median age (60.5 vs. 69.0 years, *p* = 0.50), sex (33.3% vs. 55.0% male patients, *p* = 0.14), Ann Arbor stage (stage III-IV in 58.3% vs. 48.9% of the patients, *p* = 0.52), or National Comprehensive Cancer Network-International Prognostic Index (NCCN-IPI)-scores (NCCN-IPI low, low-intermediate, high intermediate, or high in 16.7%, 33.3%, 41.7%, 8.3% vs. 8.9%, 40.1%, 35.8%, 15.2%, *p* = 0.72) between patients identified for this analysis and the other patients in our clinical cohort. Furthermore, the PFS (median PFS not reached in patients who had large lymphoma resections vs. 60.0 months in the other patients of the cohort, *p* = 0.30) and OS (median OS not reached vs. 89.0 months, *p* = 0.46) were also similar in both groups (see Table S1). The PFS-rate at 24 months of the patients with samples available for sequencing was 75%.

### 2.2. Massively Parallel Sequencing

Sequencing was successful in 93.2% of the samples resulting in 57 tumour samples (four to seven per patient) and 12 germline samples. The median tumour cell content in the lymphoma biopsies was 90% (range 40–90%, see Table S2 for tumour cell contents of all samples) and the average mean target coverage of the exons of the selected genes in the samples which were sequenced successfully was 2126x (see Table S3 for detailed sequencing quality data of all samples). Overall, non-synonymous, exonic mutations were detected in 108 (50.7%) out of 213 genes selected for NGS. The median number of mutations per patient was 29.5 (range 16–73).

In order to assess spatial genetic heterogeneity in DLBCL, we compared the mutations detected within the multiple biopsies of each of the twelve selected patients. We found at least one mutation in a gene known to be relevant for the biology of DLBCL that was equally present in all the biopsies of each patient. The allelic frequency of these mutations in genes such as *TP53*, *CARD11*, *MYD88*, *NOTCH2*, and *KMT2D* was above 30% in all biopsies. A mutation was determined to be discordant if it was present with an allelic frequency (AF) of at least 10% in one or more of the samples of one patient but not found (AF 0%) in at least one other biopsy of the same patient. This cut-off was chosen to investigate only relevant clonal changes between the different lymphoma areas. The complete list of exonic synonymous and non-synonymous mutations with an AF above 10% in at least one biopsy is shown in the Table S4, 1–12.

**Figure 1:**
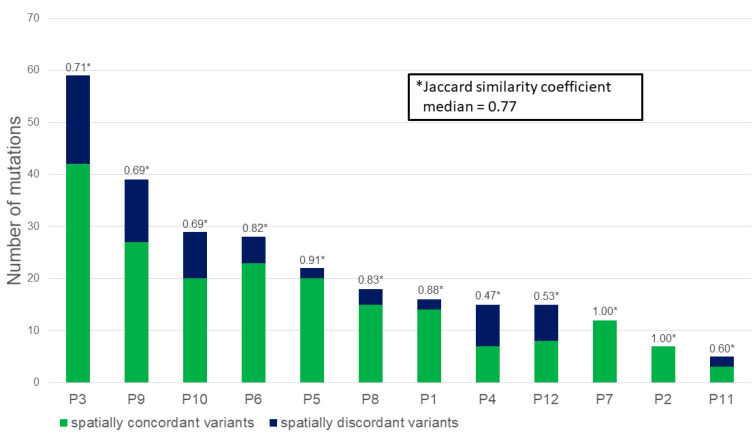
The number of non-synonymous, exonic mutations per patient with an allelic frequency of at least 10% in one of the samples. Concordant mutations that were found in all the biopsies are shown in green and discordant mutations that were not detected in at least one of the samples are shown in blue. The Jaccard similarity coefficient, indicating the similarity within the samples is given above the bar for each patient. The closer the coefficient is to one, the more homogeneous the lymphoma mass was.

The Jaccard similarity coefficient describes the similarity of a sample set and was calculated as the number of concordant mutations found in all the samples of one patient divided by the number of all mutations (concordant and discordant) per patient. The closer the coefficient is to one, the more homogenous a tumour is. Discordant mutations were found in ten out of twelve patients and the median number of discordant mutations per patient was four (range 0–17). In our sample set, the median Jaccard similarity coefficient per patient was 0.77 (range 0.47–1) (see Figure 1). A receiver operating characteristic (ROC) curve was drawn for the Jaccard coefficient and the Youden index was calculated (0.7). This was used as a cut-off to evaluate if patients with a Jaccard coefficient below or above this value had a different prognosis. However, the Jaccard coefficient had no statistically significant influence on the survival of patients in this small cohort (median OS and PFS not reached in patients with a Jaccard coefficient blow or above 0.7, PFS-rate at 24 months for patients with a Jaccard coefficient below 0.7: 100%, and PFS-rate at 24 months for patients with a Jaccard coefficient above 0.7: 57.1%; *p* = 0.11).

To further describe the discordant mutations in our patients, we calculated the 25th percentile (28%) of the AF of all the mutations in all samples and used this as a cut-off. Out of the 67 discordant mutations which were detected in ten patients, 32 mutations (47.8%) had an AF of at least 28.5% in at least one of the samples.

**Figure 2:**
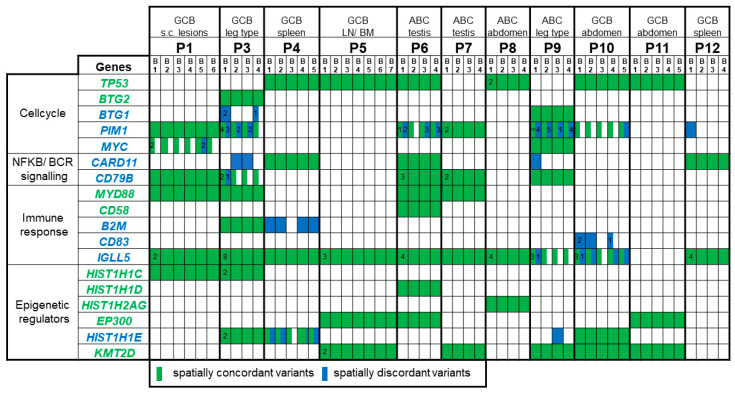
Selected genes with a known relevance for lymphoma biology. Concordant mutations which were found in all biopsies (B) are marked in green and discordant mutations which were found with an allelic frequency of at least 10% in one of the samples of a patient but not detected in at least one other are shown in blue. In some genes, several mutations were found in one patient. If multiple mutations were found, the number of mutations is given within the cell.

Figure 2 shows the mutations of the selected genes with a known biologic relevance in DLBCL. Six (50%) out of the twelve patients had at least one *TP53* mutation which was present in all the evaluated lymphoma biopsies of each patient. Five (83.3%) of the patients harbouring a *TP53* mutation are alive, with no signs of lymphoma recurrence at a median follow up of 45.0 months. Furthermore, spatially concordant mutations were found in *BTG2*, *MYD88*, *CD58*, *HIST1H1C*, *HIST1H1D*, *HIST1H2AG*, *EP300,* and *KMT2D.* However, two patients showed heterogenous *CARD11* mutations and patient 1 harboured two heterogenous *MYC* mutations, which were only found in biopsy 5 (TU5) but not in the other five lymphoma biopsies. Other spatially discordant mutations were detected for example in *BTG1, PIM1, CD79B, B2M, CD83, IGLL5,* and *HIST1H1E.* The median allelic frequency of heterogenous mutations was 29% (range 5–71%).

**Figure 3:**
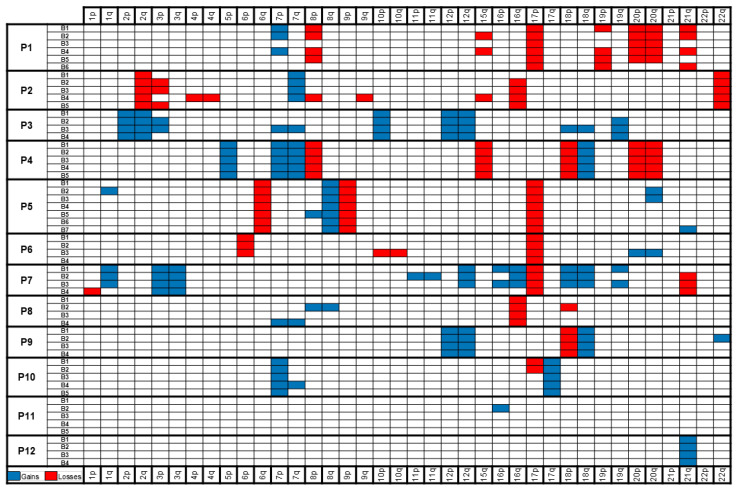
The copy number variants for all the biopsies (B) of each patient. Gains are shown in blue and losses in red.

RobustCNV revealed at least one CNV in each patient. Two patients (16.7%; patient 4 and 12) had the same alterations present in all the biopsies, while all the other patients showed at least one discordant CNV (see Figure 3). Patient 10 had a discordant deletion 17p which was only found in biopsy one and two but not in the other three biopsies of the same lymphoma mass. We were able to construct phylogenetic trees for eight of the ten patients with heterogenous CNVs.

**Figure 4:**
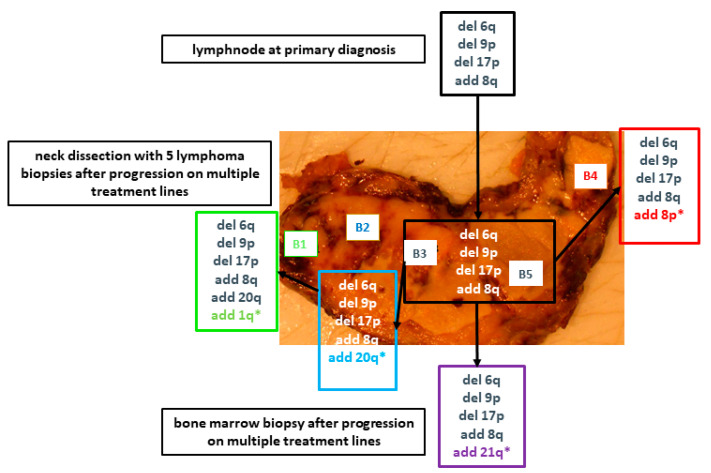
Seven biopsies of patient 5 and the detected copy number variants (CNVs). A biopsy of a single lymph node at primary diagnosis was available from this patient, which is shown at the top of the figure. The picture, in the middle of the figure, shows the specimen of the lymph node dissection which was carried out after the patient’s tumour progressed on multiple treatment lines. Shortly after the neck dissection, a bone marrow biopsy was performed and the results of the CNVs are shown at the bottom of the figure. All the samples share a complex genotype but additional CNVs (*) in some biopsies show both, spatial heterogeneity as well as clonal evolution over time.

A representative example of patient 5 is shown in Figure 4. All seven biopsies of this patient share a complex karyotype including a deletion 17p. However, biopsy 1, 2, and 4 (TU 1,2,4) as well as the bone marrow biopsy show additional alterations suggesting both clonal evolution over time as well as spatial heterogeneity in one lymphoma sample. The phylogenetic trees of the other patients are shown in the appendix (Figure S1).

## 3. Discussion

After the broad implementation of NGS techniques, the complex genetic background of DLBCL was identified by several groups [8,9,14,15]. In all of these studies, single biopsies of individual patients were analysed. However, multi-site sequencing revealed substantial intratumor heterogeneity in several different solid tumour entities which challenges the principle of targeted therapies and shows new mechanisms of drug resistance [10,11,12]. As patients with DLBCL are usually treated quickly after diagnosis from single lymph node biopsies, large lymphoma masses and multiple samples from one patient are rarely available. We characterized all the patients treated for DLBCL at our cancer center since 2004 and within this cohort we found twelve patients with available large lymphoma samples. The resections were either carried out for diagnostic reasons in patients with large abdominal tumour masses, splenomegaly or testicular swelling or therapeutically in a patient with sepsis due to cellulitis and lymphoma of the leg type. We used an NGS approach for sequencing the exons of 213 genes known to be important in lymphoma biology to investigate spatial heterogeneity in DLBCL. Pathologists took multiple biopsies from selected areas of the tumour masses with dense lymphoma infiltrates. Therefore, we were able to detect at least one mutation in each patient in a gene known to be critical for lymphoma development such as *TP53*, *MYD88*, *NOTCH2*, *KMT2D*, and *CARD11* which was present in all the samples of a single patient with similar AFs. This suggests that these mutations were early clonal events in these lymphomas and could be interpreted as driver mutations (see Table S5). Six out of twelve patients (50%) in our cohort had a *TP53* mutation which was detected in all of their biopsies. This number is higher than the 20–25% incidence rate which was reported in other cohorts and supports the finding that *TP53* mutations are more common in patients with high IPI scores [15,16]. Despite the negative prognostic influence of *TP53* mutations in patients with DLBCL which was described by different groups, the prognosis of the patients in our cohort was favourable and five out of six patients in our cohort with this alteration were still alive with no signs of lymphoma recurrence at the last point of follow-up [16,17]. In these five patients with bulky disease, the largest parts of the lymphoma manifestations were resected before the start of systemic therapy. It has been shown that in patients with early stage DLBCL, resections followed by reduced cycles of chemoimmunotherapy are feasible with good outcomes, and that in patients with intestinal lymphomas, resection prior to chemotherapy improves prognosis [18,19,20]. The results of our small cohort suggest that surgery might also play a therapeutic role in patients with a high risk, bulky disease.

Although all the patients had several mutations which were found in all of their biopsies, discordant mutations which were present in at least one biopsy with an AF above 10% but not found in one or more of the other biopsies were detected in ten out of twelve individuals in our cohort. Almost half of the discordant mutations had an AF of at least 28.5% in at least one of the biopsies in which they were detected. This suggests that these discordant mutations are not only bystander events but are present at a relevant clonal fraction. The Jaccard similarity coefficient ranged from 0.47–1 in our patients, which indicates that some DLBCLs are very homogenous while others consist of several clones with a different mutational background. Similar results were seen in nine patients with follicular lymphoma where two biopsies were taken at the same timepoint and the Jaccard similarity coefficient ranged from 0.41–0.91 [21]. In our cohort, the heterogeneity of the samples did not coincide with a higher total number of mutations and therefore a more complex genetic background in general. Although it is hard to draw definite conclusions from a small cohort, the three patients with the lowest Jaccard similarity coefficients, indicating large differences between the biopsies, belonged to the five patients with the lowest absolute number of mutations. Several of our patients showed multiple concordant and discordant mutations of *PIM1* and *IGLL5*, which are known targets of somatic hypermutation in DLBCL [13,22,23]. Although these mutations are probably not highly relevant for the biology of the disease, they help us track clonal evolution and spatial heterogeneity in our patients. Other discordant mutations were found in genes involved in the immune response, such as *CD83* and *B2M* or epigenetic regulators such as *HIST1H1E.* Interestingly, in two patients, discordant mutations were also seen in *CARD11* and *CD79B,* which are genes involved in NF-kB and B-cell receptor signaling and are known to influence the response to ibrutinib in DLBCL [24,25]. This supports the theory that single biopsies might overlook mutations with therapeutic relevance in some patients.

Similarly to what has been reported in cell lines and other patients with DLBCL there was a wide variety of CNVs found in our patients with several different chromosomes involved and no clear pattern of gains or losses [26,27]. In all except for two patients, one or more discordant CNVs were seen. Interestingly, one patient showed a discordant deletion of the short arm of chromosome 17 (del17p) leading to the loss of the tumour suppressor *TP53*, which is known to be associated with a poor prognosis [26]. Moreover, another patient had a loss of chromosome 10 in one of the biopsies, which causes a loss of the tumour suppressor *PTEN*, a known cause of resistance to T-cell mediated immunotherapy [28]. Two groups described the association of specific CNVs with GCB and non-GCB subtypes [26,27]. However, the CNVs varied between the two reports and such an association was not found within our cohort. In one patient (patient 5) where we did not only have multiple synchronous samples but also three biopsies over time, we saw a clonal evolution on the level of CNVs. This supports what we have seen in our earlier analysis where 15 out of 24 evaluable patients with a biopsy at primary diagnosis and recurrence showed large global changes on the mutational level [13].

## 4. Materials and Methods

### 4.1. Patients

In the past, we established a database of more than 380 patients who were treated for DLBCL at the Third Medical Department of the Paracelsus Medical University between 2004 and 2015 [29,30]. Out of this cohort, we selected twelve patients who had diagnostic or therapeutic resections of large lymphoma masses. Pathologists took multiple biopsies from different areas of the tumour and these formalin-fixed, paraffin-embedded (FFPE) samples were used for library construction and sequencing. The tumour cell content was evaluated in all the biopsies by haematoxylin and eosin staining, and the cell of origin was (COO) determined using the Hans classifier [31]. *MYC* translocations were analyzed using the fluorescence in situ hybridization (FISH) probe Split Signal, Code Y5410 (Dako Denmark A/S, Glostrup, Denmark). A chart-based review was applied for retrospective analysis of the patient’s clinical characteristics. Overall survival (OS) was calculated from diagnosis to the last follow-up or death from any cause, and progression free survival (PFS) was defined from diagnosis to the progression of disease or death from any cause. The Kaplan-Meier estimator was used to estimate the survival of our patients. In case a patient was lost to follow-up, we carried out telephone interviews with their general practitioners. A written informed consent was obtained from all the patients and the study was approved by the Ethics Committee of the provincial government of Salzburg, Austria (415-EP/73/127-2012).

### 4.2. Targeted Massively Parallel Sequencing

For this analysis, multiple biopsies from large lymphoma masses were used for NGS of the exons of 213 genes known to be relevant for the biology of DLBCL (for the gene list, see Table S6). Furthermore, we analysed non-tumour DNA from buccal swabs or a healthy resected tissue from each patient and compared it to the lymphoma samples to exclude germline mutations. These germline mutations are not reported in this article. SureSelect by Agilent^®^ was used for target enrichment, and the HiSeq 3000^®^ and HiSeq 2500^®^ (Illumina, Inc., San Diego, CA, USA) were used for sequencing, as previously described [32]. This approach was validated on other platforms for earlier projects and a confirmation rate above 95% was achieved [13,33]. As a minimum quality criterion, 80% of the targets of each sample had to be sequenced 30×. All reads which are reported in this manuscript were manually verified using the Integrative Genomics Viewer browser^®^. RobustCNV was used to identify the copy number variants (CNVs). This is an algorithm based on the detection of local changes in the mapping depth of the sequenced strands during targeted capture sequencing (see Supplementary Materials and Methods for details). The detected single nucleotide polymorphisms (SNPs) as well as the CNVs were compared within the multiple biopsies of each patient in order to evaluate spatial heterogeneity in DLBCL.

### 4.3. Statistical Analyses

The statistics for this manuscript were generated using the SPSS^®^ statistics software (24th version, IBM^®^, Armonk, NY, USA). The Kaplan-Meier estimator was used for survival analyses and the log-rank test was applied for statistical comparisons. For univariate analyses, the Mann-Whitney-U test and the Pearson’s Chi-squared test were used as appropriate. A *p*-value below 0.05 was considered statistically significant. Optimal cut-off values were determined by the receiver operating characteristic (ROC) calculation and Youden index analysis for OS.

## 5. Conclusions

Using an NGS approach with a selected gene panel was feasible and enabled us to depict spatial heterogeneity on a mutational level as well as on the basis of CNVs in DLBCL. This supports the results of earlier studies in solid tumour entities and follicular lymphoma [10,21]. In addition, it suggests that single biopsies in lymphoma patients might underestimate the genetic complexity of the disease and oversee possible mechanisms treatment resistance or targetable genetic variations. As multiple site sequencing is not possible in a clinical routine in most patients, the broader use of liquid biopsies with the analysis of circulating tumour DNA might be able to overcome these obstacles in the future.

## Figures and Tables

**Table 1:** Patient characteristics.

Patient	COO	Age (y)	Gender	NCCN-IPI	Sample Type	Biopsies (Number)	Treatment	PFS (mo)	OS (mo)	Status
1	GCB	41	f	low-int.	subcut. lesions	6	R-CHOP, R-EPOCH, auto-STC	47	47	alive
2	Non- GCB	80	f	high	adrenal gland, spleen, pancreas	5	1x dexa	1	1	dead
3	GCB	77	f	high-int.	lower leg	4	1x lip.doxo., rituximab	2	7	dead
4	GCB	51	m	high	spleen	5	R-CHOP	44	44	alive
5	GCB	54	m	low	LN, ND, BM	7	multiple incl. R-CHOP, DHAP, auto-SCT	1	10	dead
6	Non- GCB	82	m	high-int.	testis	4	R-DHAP	42	42	alive
7	Non- GCB	78	m	low-int.	testis	4	R-COMP	44	44	alive
8	Non- GCB	62	f	high-int.	pancreas, spleen, adrenal gland	4	R-CHOP	63	63	alive
9	Non- GCB	79	f	high-int.	subcut. lesions	4	R-COMP	23	23	alive
10	GCB	54	f	high-int.	lung, spleen	5	R-CHOP	39	39	alive
11	GCB	39	f	low	small intestine	5	R-CHOP	31	31	alive
12	GCB	60	f	low-int.	spleen	4	R-COMP	35	35	alive

COO: Cell of origin; GCB: Germinal center B-cell-like; y: Years; f: Female; m: Male; NCCN-IPI: National Comprehensive Cancer Network-International Prognostic Index; PFS: Progression free survival; OS: Overall survival; mo: Months; auto-STC: Autologous-stem cell transplantation; dexa: Dexamethasone; lip. doxo.: Liposomal doxorubicin; LN: Lymph node; ND: Neck dissection; BM: Bone marrow.

## Data Availability

The data presented in this study are available in the article and the supplementary material.

## Supplementary Materials

The following are available online at https://www.mdpi.com/2072-6694/13/4/650/s1, Figure S1: Phylogenetic trees of seven patients according to copy number variation analyses, Table S1: Comparison of patient characteristics between patients with diagnostic or therapeutic resections of large lymphoma masses and patients with single diagnostic biopsies in the cohort of patients with DLBCL treated at the Third Medical Department of the Paracelsus Medical University, Table S2: Tumor cell content of all lymphoma samples evaluated by haematoxylin and eosin staining, Table S3: Sequencing quality data, Table S4, 1–12: Somatic, exonic mutations detected in patients 1–12, Table S5: Suggested driver mutations with stable allelic frequencies (AF) across all the biopsies (B) of a patient, Table S6: Selected genes for exon sequencing.
