# Supplementary material for: Spatial Heterogeneity in Large Resected Diffuse Large B-Cell Lymphoma Bulks Analysed by Massively Parallel Sequencing of Multiple Synchronous Biopsies
Source: Cancers (Basel). 2021 Feb 6;13(4):650. doi: 10.3390/cancers13040650 (PMC7914762; doi:10.3390/cancers13040650)
Supplement: Supplementary file 1 [file cancers-13-00650-s001.pdf]

cancers

MDPI

Supplementary Materials

Spatial Heterogeneity in Large Resected Diffuse Large B-Cell Lymphoma Bulks Analysed by Massively Parallel Sequencing of Multiple Synchronous Biopsies

Teresa Magnes, Sandro Wagner, Aaron R. Thorner, Daniel Neureiter, Eckhard Klieser, Gabriel Rinnerthaler, Lukas Weiss, Florian Huemer, Konstantin Schlick, Nadja Zaborsky, Markus Steiner, Richard Greil, Alexander Egle and Thomas Melchardt

Citation: Magnes, T.; Wagner, S.; Thorner, A.R.; Neureiter, D.; Klieser, E.; Rinnerthaler, G.; Weiss, L.; Huemer, F.; Schlick, K.; Zaborsky, N.; et al. Spatial Heterogeneity in Large Resected Diffuse Large B-Cell Lymphoma Bulks Analysed by Massively Parallel Sequencing of Multiple Synchronous Biopsies. *Cancers* **2021**, *13*, 650. https://10.3390/cancers13040650

Academic Editor: Paul Murray

Received: 11 January 2021
Accepted: 3 February 2021
Published: 6 February 2021

Publisher's Note: MDPI stays neutral with regard to jurisdictional claims in published maps and institutional affiliations.

Copyright: © 2021 by the authors. Submitted for possible open access publication under the terms and conditions of the Creative Commons Attribution (CC BY) license (http://creativecommons.org/licenses/by/4.0/).

1. Supplementary Materials and Methods

1.1. Sequencing Library Preparation

Genomic DNA was isolated from formalin-fixed paraffin-embedded (FFPE) samples using the QIAamp DNA FFPE Tissue Kit and from blood using QIAamp DNA Blood Mini Kit (Qiagen, Venlo, Netherlands). Two hundred nanograms (ng) of genomic DNA from FFPE samples and 100 ng from peripheral blood mononuclear cells (PBMCs) was fragmented (Covis sonication) to 250 base pairs and purified with Agentcourt AMPure XP® beads. The DNA was then ligated to specific adaptors (SPRIworks. Beckman-Coulter®, Brea, CA, USA), and a library yield of >250 ng was assessed to be successful. The sample quality was evaluated before and after fragmentation by an Agilent TapeStation (Agilent Technologies, Santa Clara, CA, USA) and libraries were quantified using an Illumina MiSeq (Illumina, San Diego, CA, USA). In total, seven captures were performed, and the Agilent SureSelect hybrid capture was used to enrich the selected genes. RNA probes that are 120 nt in length and homologous to the regions of interest were synthesized. These probes were used to "capture" DNA from the whole genome libraries that were created, thereby enriching the selected genes. Five captures were sequenced on three lanes of the Illumina HiSeq 3000 and two captures were sequenced over two lanes of the Illumina HiSeq 2500.

1.2. Pre-Analysis Processing

Picard tools were used to de-convolute (de-multiplex) and sort the pooled sample reads (see http://broadinstitute.github.io/picard/picard-metric-definitions.html for details).

The sequence b37 edition from the Human Genome Reference Consortium using bwa aln (http://bio-bwa.sourceforge.net/bwa.shtml) was used as a reference and the following parameters were applied: "-q (Parameter for read trimming. BWA trims a read down to argmax_x{\sum_{i=x+1}^l(INT-q_i)} if q_l<INT where l is the original read length) 5-l (Take the first INT subsequence as seed. If INT is larger than the query sequence, seeding will be disabled. For long reads, this option is typically ranged from 25 to 35 for '-k 2'.) 32 -k (Maximum edit distance in the seed) 2 -o (Maximum occurrences of a read for pairing. A read with more occurrneces will be treated as a single-end read. Reducing this parameter helps faster pairing.) 1". Duplicate reads were identified and removed using the Picard tools.

1.3. Quality Control for Sequencing and Variant Analysis

For each sample at least 80% of the targets had to be sequenced 30×. The MuText v1.1.4 was used for the analysis of Single nucleotide variants (SNV), and mutations were annotated by the Variant Effect Predictor (VEP). The SomaticIndelDetector tool which is part of the GATK was used for indel calling.

1.4. Copy Number Variants

RobustCNV is an algorithm to analyse copy number variants (CNVs) developed at the Center for Cancer Genome Discovery (CCGD) at the Dana Faber Cancer Institute in Boston. Changes in copy number are detected through localized changes in the mapping depth of sequenced reads. The algorithm includes a normalization step where robust regression is used to compare the observed tumour mapping depth against a panel of normals (PON) sampled with the same capture bait set. The detected values are normalized against predicted values and given as log2ratios. A loess fit is applied to remove GC bias. Finally, log2ratios are centered on segments determined to be diploid based on the allele fraction of heterozygous SNPs in the targeted panel.

Circular Binary Segmentation (Olshen et al., 2004) from the DNAcopy Bioconductor package was applied to segment the normalized coverage data. Lastly, it is determined whether the segments have additional copies (gain), less copies (loss) or normal-copy numbers based on a cutoff developed from the within-segment standard deviation of post-normalized mapping depths and a tuning parameter which was set based on comparisons to array-CGH calls in separate validation experiments.

2. Supplementary Figure

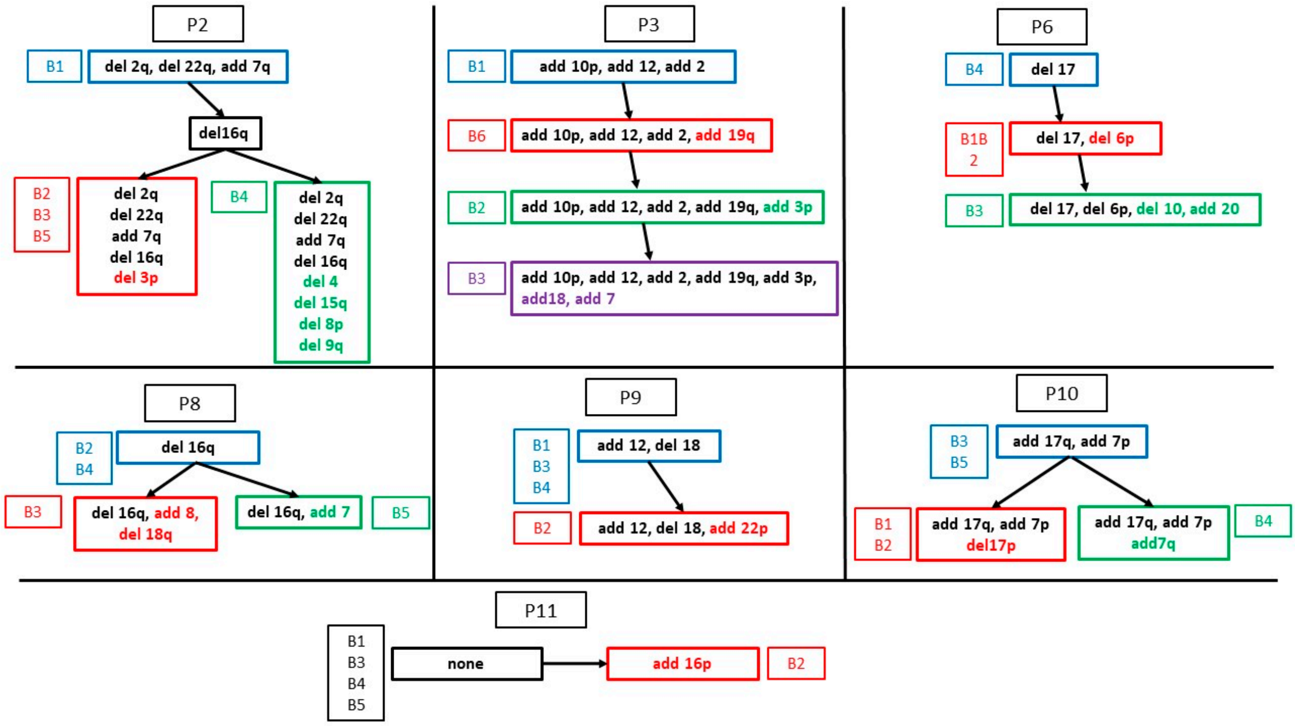

Figure S1. Phylogenetic trees of seven patients according to copy number variation analyses.

3. Supplementary Tables

Table S1. Comparison of patient characteristics between patients with diagnostic or therapeutic resections of large lymphoma masses and patients with single diagnostic biopsies in the cohort of patients with DLBCL treated at the Third Medical Department of the Paracelsus Medical University.

Patient Characteristic	Patients with Large Lymphoma Resections (n = 12)	Patients with Diagnostic Biopsies (n = 331)	p-Value
median age (years)	60.5	69.0	0.50 [1]

sex (male patients)	33.3%	55.0%	0.14 [2]
Ann Arbor stage (stage III-IV)	58.3%	48.9%	0.52 [2]
NCCN-IPI stage			
low	16.7%	8.9%	
low-intermediate	33.3%	40.1%	0.72 [2]
high-intermediate	41.7%	35.8%	
high	9.3%	15.2%	
median PFS (months)	not reached	60.0	0.30 [3]
median OS (months)	not reached	89.0	0.46 [3]

1 = Mann-Whitney-U test, 2 = Pearson's Chi-squared test, 3 = Log Rank test.

Table 2. Tumor cell content of all lymphoma samples evaluated by haematoxylin and eosin staining. (B = biopsy, X = biopsy was not available for this patient).

Patient	B1	B2	B3	B4	B5	B6	B7
1	90%	40%	80%	70%	80%	80%	X
2	70%	90%	90%	90%	70%	X	X
3	80%	90%	90%	90%	X	X	X
4	80%	90%	80%	90%	90%	X	X
5	90%	90%	90%	90%	80%	80%	80%
6	90%	70%	80%	70%	X	X	X
7	90%	90%	80%	80%	X	X	X
8	70%	70%	50%	50%	X	X	X
9	90%	90%	80%	80%	X	X	X
10	90%	90%	90%	90%	90%	X	X
11	90%	80%	70%	80%	80%	X	X
12	90%	90%	90%	90%	90%	X	X

Table 3. Sequencing quality data. (B = biopsy, GL = germline).

Patient/Biopsy	Number of PF Reads	% Selected Bases	Mean Target Coverage (x)	% Duplication	% Target Bases Covered 30×
1, B1	37,306,756	46.3	278.6	46.5	97.9
1, B2	23,864,954	50.5	186.0	45.6	96.2
1, B3	23,923,976	49.5	176.5	47.4	96.4
1, B4	47,389,054	47.7	370.4	43.9	98.5
1, B5	39,559,710	47.9	323.1	42.3	98.2
1, B6	43,504,098	45.1	322.6	43.2	98.2
1, GL	38,447,388	49.2	410.3	29.4	99.1
2, B1	31,572,496	37.2	136.5	62.4	96.6
2, B2	25,939,158	28.0	95.4	56.3	92.6
2, B3	23,163,556	37.8	92.5	65.7	93.6
2, B4	35,167,090	33.0	164.4	52.6	97.5
2, B5	30,526,784	29.2	99.7	63.0	95.1
2, GL	24,194,860	39.9	139.5	52.6	96.4
3, B1	25,315,146	42.9	138.6	57.5	96.0
3, B2	43,354,280	36.7	249.1	48.4	98.5
3, B3	31,487,818	41.3	183.8	54.5	97.0
3, B4	28,383,320	33.5	127.2	56.4	95.5
3, GL	34,444,040	48.5	335.8	30.8	99.0
4, B1	30,653,436	37.2	139.3	60.0	97.1

4, B2	24,683,826	46.0	150.6	56.6	97.0
4, B3	45,738,986	29.5	167.0	60.1	97.6
4, B4	35,896,686	41.6	196.5	58.1	97.9
4, B5	36,844,182	33.3	129.9	65.1	95.9
4, GL	35,946,266	48.7	353.4	34.0	98.9
5, B1	25,732,688	41.1	147.7	54.5	94.6
5, B2	32,203,526	45.6	179.1	61.2	95.7
5, B3	34,177,940	44.8	178.9	62.1	96.4
5, B4	39,371,550	46.3	216.7	61.9	96.0
5, B5	44,363,076	43.4	315.9	46.4	97.8
5, B6	28,542,600	42.7	188.5	49.2	95.8
5, B7	44,516,136	41.3	311.8	45.3	98.8
5, GL	38,776,202	51.3	439.4	28.7	99.1
6, B1	24,103,486	26.0	90.1	46.8	87.9
6, B2	49,352,054	36.9	253.9	52.1	98.0
6, B3	31,437,998	37.9	183.2	47.7	96.3
6, B4	18,489,216	38.6	102.5	48.2	85.3
6, GL	28,737,418	49.9	299.3	31.3	98.7
7, B1	22,633,848	32.0	103.6	51.5	88.4
7, B2	23,257,472	36.5	123.3	52.2	93.1
7, B3	34,761,526	26.5	110.3	59.5	91.9
7, B4	40,684,320	21.1	103.1	60.2	91.9
7, GL	40,946,958	49.8	442.9	29.2	99.1
8, B1	27,004,548	24.6	79.7	62.1	85.5
8, B2	28,363,520	23.0	70.9	63.9	86.9
8, B3	37,961,030	28.5	126.0	62.8	93.9
8, B4	22,825,466	31.5	96.7	58.1	83.8
8, GL	41,510,746	50.8	452.2	29.0	99.1
9, B1	23,523,380	31.3	98.0	53.2	91.4
9, B2	28,043,856	38.2	134.3	56.1	94.8
9, B3	31,793,496	37.9	166.6	54.6	96.1
9, B4	27,745,390	38.5	155.8	51.0	95.4
9, GL	32,387,412	49.2	337.7	30.3	98.7
10, B1	55,282,362	40.4	316.8	53.9	98.7
10, B2	52,949,152	40.3	310.5	53.8	98.1
10, B3	39,717,682	46.2	247.0	56.8	98.1
10, B4	39,667,754	43.4	241.6	55.4	97.4
10, B5	33,159,632	47.6	225.3	53.2	98.3
10, GL	35,192,292	51.2	404.1	28.8	98.9
11, B1	33,835,906	42.9	220.9	52.6	95.9
11, B2	32,338,006	48.0	249.5	49.5	96.8
11, B3	29,199,986	40.4	139.2	60.8	97.3
11, B4	34,806,632	44.9	211.5	55.4	98.0
11, B5	34,720,056	37.2	151.4	61.2	97.5
11, GL	35,854,370	48.4	361.9	31.2	98.9
12, B1	35,530,622	35.3	178.2	51.8	97.3
12, B2	37,663,780	36.5	189.1	53.7	97.5
12, B3	41,335,912	34.2	201.1	53.0	97.5
12, B4	33,660,222	34.4	160.1	54.3	96.9
12, GL	34,643,634	51.3	383.0	29.0	99.0

Table S4. 1. Somatic, exonic mutations detected in patient 1. (Chr. = chromosome, AF = allelic frequency, Cov. = coverage, B = biopsy).

Chr.	Variant Classification	Gene	cDNA Change	AF B1	CCov. B1	AF B2	CCov. B2	AF B3	CCov. B3	AF B4	Cov. B4	AF B5	Cov. B5	AF B6	Cov. B6
4	Missense	*FGFRL1*	c.1085C>A	0.15	68	0.21	43	0.16	43	0.12	109	0.08	107	0.11	97

22	Missense	*IGLL5*	c.131C>T	0.88	94	0.79	75	0.76	68	0.74	103	0.77	93	0.82	104
22	Missense	*IGLL5*	c.157G>A	0.87	113	0.81	74	0.73	74	0.71	101	0.78	89	0.82	109
6	Missense	*HIST1H1C*	c.476A>G	0.47	342	0.45	280	0.47	229	0.57	450	0.45	380	0.43	412
18	Missense	*KLHL14*	c.800C>T	0.45	311	0.44	228	0.42	187	0.49	329	0.45	322	0.39	293
6	Missense	*PIM1*	c.499G>C	0.47	265	0.37	166	0.39	139	0.47	283	0.49	252	0.47	269
3	Stop_Lost	*MYD88*	c.478T>C	0.43	300	0.37	234	0.41	229	0.46	412	0.45	394	0.42	355
17	Missense	*CD79B*	c.589T>C	0.42	175	0.41	139	0.35	165	0.37	242	0.45	224	0.43	230
8	Missense	*MYC*	c.38C>T	0.53	380	0.52	264	0.47	229	0.44	448	0.50	394	0.51	434
8	Missense	*MYC*	c.563C>T	0.00	0	0.00	0	0.00	0	0.00	0	0.22	487	0.00	0
8	Missense	*MYC*	c.566C>T	0.00	0	0.00	0	0.00	0	0.00	0	0.22	483	0.00	0
8	Missense	*MYC*	c.895G>A	0.50	409	0.44	306	0.46	295	0.45	607	0.45	444	0.48	421
8	Missense	*BAI1*	c.4696T>C	0.48	29	0.44	16	0.35	17	0.32	31	0.40	30	0.32	37
3	Missense	*TBL1XR1*	c.1337A>C	0.44	473	0.41	328	0.41	306	0.40	596	0.42	541	0.44	563
2	Missense	*TTN*	c.87826A>C	0.42	431	0.39	346	0.40	313	0.44	673	0.35	573	0.47	547
2	Missense	*ZNF804A*	c.27C>G	0.47	331	0.42	233	0.35	187	0.43	380	0.44	339	0.46	364

Table S4. 2. Somatic, exonic mutations detected in patient 2.

Chr.	Variant Classification	Gene	cDNA Change	AF B1	Cov. B1	AF B2	Cov. B2	AF B3	Cov. B3	AF B4	Cov. B4	AF B5	Cov. B5
19	synonymous	*MUC16*	c.5109C>T	0.00	0	0.00	0	0.13	90	0.27	96	0.00	0
12	Missense	*CDKN1B*	c.524C>T	0.25	255	0.20	142	0.19	130	0.33	274	0.20	189
9	synonymous	*MPDZ*	c.5409T>G	0.17	173	0.23	88	0.26	103	0.21	247	0.19	128
9	Missense	*MPDZ*	c.4093G>C	0.27	177	0.27	104	0.17	98	0.28	239	0.20	134
6	Nonsense	*HLA-C*	c.232C>T	0.32	228	0.29	190	0.25	169	0.38	154	0.19	138
6	Nonsense	*HLA-B*	c.324C>G	0.40	30	0.24	21	0.18	28	0.65	17	0.28	18
17	Missense	*IKZF3*	c.485T>G	0.27	175	0.21	130	0.19	99	0.41	196	0.28	121
X	Splice_Donor	*UBE2A*	c.125+2T>C	0.22	209	0.17	211	0.22	169	0.29	163	0.16	133
1	Frameshift	*NOTCH2*	c.6909delC	0.38	102	0.42	71	0.37	63	0.53	120	0.39	87

Table S4. 3. Somatic, exonic mutations detected in patient 3.

Chr.	Variant Classification	Gene	cDNA Change	AF B1	Cov. B1	AF B2	Cov. B2	AF B3	Cov. B3	AF B4	Cov. B4
6	synonymous	*IRF4*	c.738G>T	0.78	107	0.89	201	0.80	151	0.65	102
7	Missense	*CARD11*	c.368G>A	0.00	0	0.06	145	0.60	262	0.00	0
17	Missense	*P2RX5*	c.106C>A	0.36	160	0.44	313	0.43	292	0.41	210
17	synonymous	*P2RX5*	c.93G>A	0.45	154	0.42	293	0.46	281	0.34	215
7	Missense	*ACTB*	c.158A>C	0.35	117	0.45	174	0.56	249	0.41	138
19	Missense	*CD70*	c.500T>A	0.71	123	0.82	232	0.86	236	0.80	198
12	Splice_Region	*ETV6*	c.33G>A	0.53	154	0.53	296	0.56	247	0.57	148
12	Splice_Donor	*ETV6*	c.33+1G>A	0.54	155	0.53	298	0.56	250	0.57	148
12	Nonsense	*CDKN1B*	c.157G>T	0.28	221	0.28	463	0.27	463	0.24	276
X	Nonsense	*TMSB4X*	c.118C>T	0.41	102	0.49	308	0.44	116	0.27	70
9	Nonsense	*CDKN2A*	c.238C>T	0.49	81	0.49	267	0.50	208	0.41	267
14	synonymous	*TRAJ33*	c.42C>T	0.36	145	0.48	300	0.48	132	0.40	73
22	Missense	*IGLL5*	c.56G>A	0.24	70	0.30	172	0.36	143	0.21	112
22	synonymous	*IGLL5*	c.82C>T	0.19	68	0.20	162	0.29	145	0.13	116
22	Missense	*IGLL5*	c.95C>G	0.21	70	0.16	146	0.23	142	0.13	119
22	Missense	*IGLL5*	c.115C>G	0.20	79	0.16	146	0.22	149	0.12	130
22	synonymous	*IGLL5*	c.135G>A	0.00	0	0.19	168	0.00	0	0.21	144
22	synonymous	*IGLL5*	c.186C>T	0.31	58	0.29	159	0.27	136	0.29	124
22	Missense	*IGLL5*	c.206G>A	0.42	43	0.56	135	0.55	114	0.49	87
22	Splice_Donor	*IGLL5*	c.206+1G>C	0.43	42	0.57	134	0.56	113	0.51	85
22	Missense	*IGLL5*	c.260G>A	0.37	98	0.39	258	0.31	214	0.30	125
22	Missense	*IGLL5*	c.505C>G	0.42	125	0.38	234	0.41	220	0.33	120
22	Missense	*IGLL5*	c.614A>C	0.36	100	0.46	174	0.46	180	0.39	72

6	Missense	*HIST1H1C*	c.199G>A	0.69	162	0.83	255	0.75	203	0.62	98
6	Missense	*HIST1H1C*	c.169G>C	0.70	138	0.82	253	0.75	206	0.57	87
6	Missense	*HIST1H1E*	c.193_195del GCGinsACA	0.36	156	0.44	283	0.39	271	0.35	188
6	Missense	*HIST1H1E*	c.331G>A	0.35	130	0.39	197	0.35	203	0.36	171
18	Missense	*KLHL14*	c.76C>T	0.47	45	0.00	0	0.00	0	0.00	0
6	synonymous	*PIM1*	c.73C>T	0.41	99	0.44	269	0.44	265	0.37	276
6	Splice_Region	*PIM1*	c.83-4C>T	0.33	81	0.40	184	0.41	162	0.37	167
6	Missense	*PIM1*	c.83G>A	0.41	81	0.45	183	0.43	166	0.39	169
6	Missense	*PIM1*	c.97C>T	0.77	90	0.85	196	0.80	171	0.77	163
6	synonymous	*PIM1*	c.237G>A	0.34	77	0.40	203	0.38	169	0.28	134
6	Splice_Donor	*PIM1*	c.240+1G>A	0.35	77	0.38	200	0.39	175	0.26	129
6	Splice_Region	*PIM1*	c.241-3C>T	0.45	101	0.00	0	0.00	0	0.00	0
6	synonymous	*PIM1*	c.357C>T	0.37	121	0.00	0	0.00	0	0.00	0
6	Missense	*PIM1*	c.373C>T	0.00	0	0.00	0	0.35	259	0.00	0
6	synonymous	*PIM1*	c.378G>A	0.28	115	0.00	0	0.00	0	0.00	0
6	Nonsense	*PIM1*	c.379C>T	0.30	116	0.11	238	0.00	0	0.00	0
6	synonymous	*PIM1*	c.402G>A	0.27	115	0.41	230	0.41	268	0.43	229
6	Missense	*PIM1*	c.496C>G	0.00	0	0.05	218	0.49	262	0.00	0
6	Splice_Region	*PIM1*	c.607+5G>C	0.44	57	0.00	0	0.00	0	0.00	0
3	Missense	*MYD88*	c.719T>C	0.42	113	0.58	230	0.56	162	0.36	89
15	Nonsense	*B2M*	c.20T>G	0.77	107	0.85	194	0.86	197	0.68	116
11	synonymous	*MPEG1*	c.1452C>T	0.39	203	0.43	304	0.40	215	0.40	115
8	Missense	*TOX*	c.102G>C	0.00	0	0.31	183	0.00	0	0.41	132
17	Missense	*CD79B*	c.589T>A	0.51	87	0.47	135	0.41	142	0.35	94
17	Splice_Region	*CD79B*	c.433+3G>T	0.40	99	0.40	179	0.37	174	0.39	116
17	Missense	*CD79B*	c.427G>C	0.40	111	0.00	0	0.00	0	0.00	0
17	Missense	*CD79B*	c.193C>T	0.39	134	0.43	248	0.44	236	0.40	183
14	Missense	*ZFP36L1*	c.494G>A	0.00	0	0.38	269	0.44	265	0.41	202
14	Missense	*ZFP36L1*	c.137G>C	0.38	200	0.00	0	0.00	0	0.00	0
X	Missense	*TAF1*	c.3838G>A	0.32	120	0.31	214	0.40	158	0.35	118
7	Missense	*PCLO*	c.12029A>G	0.33	184	0.41	254	0.57	237	0.43	97
7	Missense	*PCLO*	c.12022A>C	0.32	181	0.42	255	0.57	242	0.42	98
7	synonymous	*PCLO*	c.10545C>T	0.39	161	0.42	276	0.55	259	0.31	112
12	Splice_Region	*BTG1*	c.148+5G>A	0.38	97	0.00	0	0.00	0	0.00	0
12	synonymous	*BTG1*	c.139C>T	0.24	98	0.28	264	0.29	264	0.27	289
12	synonymous	*BTG1*	c.109C>T	0.32	105	0.29	292	0.27	272	0.29	307
12	synonymous	*BTG1*	c.108G>A	0.00	0	0.31	294	0.29	273	0.28	307
12	synonymous	*BTG1*	c.90G>A	0.00	0	0.17	303	0.00	0	0.00	0
12	Missense	*BTG1*	c.76C>G	0.00	0	0.00	0	0.00	0	0.12	290
12	Missense	*BTG1*	c.50C>A	0.27	104	0.00	0	0.00	0	0.00	0
12	synonymous	*BTG1*	c.42G>A	0.00	0	0.30	254	0.29	224	0.25	237
2	synonymous	*DUSP2*	c.573G>A	0.31	126	0.00	0	0.00	0	0.00	0
6	Nonsense	*PRDM1*	c.180C>G	0.00	0	0.57	258	0.00	0	0.25	63
7	Missense	*LRRN3*	c.1363T>A	0.38	175	0.42	275	0.29	245	0.38	106
12	synonymous	*DTX1*	c.33T>A	0.32	91	0.42	198	0.38	182	0.27	179
12	Missense	*DTX1*	c.47G>C	0.52	99	0.40	210	0.45	193	0.47	192
12	Missense	*DTX1*	c.76_78del GTGinsATA	0.36	108	0.28	247	0.33	238	0.32	244
12	synonymous	*DTX1*	c.102C>T	0.30	128	0.42	266	0.35	306	0.27	290
12	Nonsense	*DTX1*	c.120C>A	0.31	135	0.14	278	0.28	337	0.29	309
12	Missense	*DTX1*	c.134G>A	0.29	136	0.29	278	0.28	354	0.24	299
12	Missense	*DTX1*	c.211C>G	0.25	118	0.18	201	0.24	278	0.28	214
12	Missense	*DTX1*	c.217A>G	0.35	112	0.29	205	0.34	271	0.31	204
X	Nonsense	*UBE2A*	c.49C>T	0.41	70	0.42	150	0.50	149	0.23	173
6	Frameshift	*TNFAIP3*	c.102_103insG	0.00	0	0.11	327	0.00	0	0.00	0
5	Missense	*EBF1*	c.721G>T	0.40	196	0.45	416	0.39	196	0.37	126

5	Missense	*EBF1*	c.194C>T	0.37	138	0.00	0	0.00	0	0.00	0
1	Missense	*BTG2*	c.103C>T	0.39	96	0.42	246	0.43	209	0.35	207
1	synonymous	*BTG2*	c.423G>A	0.30	96	0.39	211	0.40	250	0.33	187
1	synonymous	*ITPKB*	c.432C>T	0.36	107	0.45	219	0.36	238	0.37	234

Table S4. 4. Somatic, exonic mutations detected in patient 4.

Chr.	Variant Classification	Gene	cDNA Change	AF B1	Cov. B1	AF B2	Cov. B2	AF B3	Cov. B3	AF B4	Cov. B4	AF B5	Cov. B5
X	Missense	*P2RY8*	c.751G>A	0.54	359	0.69	276	0.00	0	0.70	249	0.65	288
X	Inframe_Del	*P2RY8*	c.287_301delGCAACGTGGTGACCG	0.00	0	0.00	0	0.10	134	0.00	0	0.00	0
X	Frameshift	*P2RY8*	c.262_284delCACTGGGTATTCGGGGTGCTGCT	0.00	0	0.00	0	0.10	137	0.00	0	0.00	0
8	Missense	*MYOM2*	c.911C>T	0.47	249	0.70	199	0.60	237	0.66	239	0.70	227
7	Inframe_Del	*CARD11*	c.1864_1866delTCC	0.65	190	0.71	189	0.70	336	0.72	308	0.75	272
17	Missense	*TP53*	c.526T>C	0.32	361	0.44	289	0.38	314	0.38	403	0.40	426
X	Splice_Region	*TMSB4X*	c.100+5G>A	0.20	94	0.00	0	0.00	0	0.00	0	0.00	0
X	Missense	*TMSB4X*	c.114G>C	0.00	0	0.37	71	0.00	0	0.00	0	0.00	0
22	Missense	*IGLL5*	c.182C>G	0.56	101	0.74	84	0.58	95	0.79	124	0.78	122
22	synonymous	*IGLL5*	c.195C>T	0.55	92	0.74	90	0.57	88	0.82	120	0.74	116
6	Missense	*HIST1H1E*	c.193G>C	0.34	287	0.40	223	0.53	160	0.42	347	0.38	327
6	Missense	*HIST1H1E*	c.500C>G	0.37	126	0.43	94	0.00	0	0.46	194	0.36	202
6	Frameshift	*HLA-A*	c.595delG	0.21	209	0.31	156	0.36	132	0.31	242	0.32	260
15	Frameshift	*B2M*	c.302_305delGTGT	0.34	121	0.55	146	0.00	0	0.71	188	0.47	64
6	Splice_Donor	*COL12A1*	c.8941+1G>A	0.00	0	0.00	0	0.11	196	0.00	0	0.00	0
18	synonymous	*SALL3*	c.159C>T	0.11	797	0.11	709	0.21	503	0.00	0	0.07	747
1	Missense	*OBSCN*	c.19895C>T	0.30	146	0.40	89	0.48	133	0.46	173	0.35	185

Table S4. 5. Somatic, exonic mutations detected in patient 5.

Chr.	Variant Classification	Gene	cDNA Change	AF B1	Cov. B1	AF B2	Cov. B2	AF B3	Cov. B3	AF B4	Cov. B4	AF B5	Cov. B5	AF B6	Cov. B6	AF B7	Cov. B7
17	Missense	*TP53*	c.517G>A	0.81	125	0.92	143	0.85	157	0.72	168	0.82	198	0.70	149	0.53	225
5	Missense	*DNAH5*	c.11986C>T	0.37	139	0.45	113	0.44	126	0.43	117	0.44	266	0.40	174	0.29	258
22	Missense	*IGLL5*	c.35C>T	0.54	83	0.62	60	0.50	42	0.42	36	0.38	68	0.37	65	0.39	150
22	Missense	*IGLL5*	c.85C>G	0.58	76	0.77	44	0.56	41	0.45	40	0.48	52	0.45	65	0.48	132
22	Missense	*IGLL5*	c.176G>C	0.42	55	0.60	43	0.48	27	0.20	35	0.50	42	0.40	53	0.31	94
22	synonymous	*IGLL5*	c.195C>T	0.31	61	0.46	39	0.23	22	0.21	29	0.35	43	0.30	46	0.21	97
22	Missense	*EP300*	c.4400A>T	0.42	219	0.46	289	0.39	264	0.41	359	0.48	453	0.44	242	0.34	474
12	Frameshift	*KMT2D*	c.3704dupG	0.60	334	0.61	515	0.60	420	0.53	453	0.57	579	0.54	403	0.50	549
12	Frameshift	*KMT2D*	c.1940dupC	0.25	319	0.19	360	0.17	352	0.31	354	0.26	468	0.15	369	0.24	576

18	Missense	*BCL2*	c.467 T>C	0.43	269	0.69	550	0.54	397	0.56	443	0.55	507	0.43	403	0.38	468
18	Missense	*BCL2*	c.338 C>G	0.41	140	0.69	358	0.52	281	0.54	276	0.58	301	0.39	242	0.34	319
18	Missense	*BCL2*	c.140 G>A	0.44	94	0.70	217	0.63	176	0.55	161	0.62	221	0.47	144	0.37	209
4	synonymous	*WDFY3*	c.612 G>A	0.47	134	0.54	125	0.42	91	0.40	161	0.50	239	0.39	151	0.34	223
8	Nonsense	*CSMD3*	c.2587 G>T	0.55	221	0.61	200	0.66	235	0.59	211	0.72	333	0.56	216	0.51	408
12	Missense	*BCL7A*	c.33 G>C	0.44	41	0.62	50	0.53	51	0.38	65	0.38	74	0.38	45	0.37	78
6	Missense	*SGK1*	c.501 C>G	0.82	72	0.96	78	0.87	76	0.70	105	0.78	137	0.71	84	0.45	159
6	synonymous	*SGK1*	c.486 G>A	0.80	70	0.97	73	0.88	74	0.71	111	0.77	132	0.70	81	0.45	161
6	Splice_ Region	*SGK1*	c.437+5 G>T	0.35	26	0.73	26	0.46	26	0.47	62	0.56	68	0.63	51	0.19	141
6	Splice_ Donor	*SGK1*	c.437+2 T>C	0.30	23	0.75	28	0.52	29	0.48	61	0.58	71	0.62	50	0.19	144
6	Missense	*SGK1*	c.418 A>C	0.58	43	0.85	47	0.73	48	0.65	104	0.75	126	0.68	81	0.28	186
6	Frameshift	*SGK1*	c.372 delG	0.70	93	0.94	129	0.86	118	0.79	205	0.87	293	0.81	164	0.46	314
2	Missense	*LRP1B*	c.10298 A>C	0.46	140	0.45	192	0.48	159	0.43	239	0.48	349	0.47	163	0.29	287
3	Missense	*MED12L*	c.4100 C>G	0.00	0	0.00	0	0.00	0	0.00	0	0.00	0	0.20	204	0.00	0
1	Missense	*TCHH*	c.2957 G>T	0.00	0	0.00	0	0.00	0	0.00	0	0.00	0	0.00	0	0.27	691
3	Splice_ Acceptor	*TBL1XR1*	c.865 -2A>G	0.36	91	0.45	83	0.54	127	0.46	90	0.42	204	0.40	80	0.40	209

Table S4. 6. Somatic, exonic mutations detected in patient 6.

Chr.	Variant Classification	Gene	cDNA Change	AF B1	Cov. B1	AF B2	Cov. B2	AF B3	Cov. B3	AF B4	Cov. B4
6	synonymous	*IRF4*	c.111C>T	0.50	169	0.49	312	0.75	229	0.51	128
7	Missense	*CARD11*	c.383C>T	0.15	54	0.18	498	0.24	356	0.16	232
7	Missense	*ACTB*	c.98C>T	0.00	0	0.00	0	0.29	141	0.00	0
19	Missense	*CD70*	c.500T>G	0.64	103	0.60	665	0.85	550	0.68	325
17	Splice_Acceptor	*TP53*	c.376-1G>A	0.40	72	0.47	396	0.75	276	0.47	196
22	synonymous	*IGLL5*	c.82C>T	0.29	72	0.41	356	0.57	265	0.37	135
22	Missense	*IGLL5*	c.95C>A	0.11	71	0.18	355	0.26	266	0.15	130
22	synonymous	*IGLL5*	c.115C>T	0.14	65	0.18	368	0.25	256	0.17	146
22	Missense	*IGLL5*	c.126G>A	0.14	65	0.18	370	0.27	256	0.17	148
22	Missense	*IGLL5*	c.161C>T	0.24	78	0.36	332	0.51	266	0.32	114
22	synonymous	*IGLL5*	c.183C>T	0.23	75	0.39	311	0.50	243	0.35	97
22	Missense	*IGLL5*	c.205A>C	0.15	65	0.17	282	0.25	223	0.20	85
6	synonymous	*HIST1H1E*	c.234C>T	0.38	92	0.34	675	0.53	494	0.29	185
6	Missense	*HIST1H1D*	c.370G>A	0.23	65	0.11	328	0.23	243	0.19	62
6	Missense	*HLA-A*	c.562T>A	0.56	91	0.61	393	0.76	298	0.56	188
6	synonymous	*PIM1*	c.51C>T	0.05	288	0.00	0	0.27	433	0.12	136
6	Missense	*PIM1*	c.83G>A	0.00	0	0.00	0	0.29	286	0.08	83
6	Missense	*PIM1*	c.202C>G	0.07	122	0.00	0	0.22	268	0.08	115
6	synonymous	*PIM1*	c.237G>A	0.09	97	0.00	0	0.23	268	0.11	111
6	Splice_Region	*PIM1*	c.240+8C>T	0.07	81	0.00	0	0.23	261	0.10	107

6	Missense	*PIM1*	c.290G>A	0.46	71	0.44	420	0.52	377	0.40	124
6	synonymous	*PIM1*	c.549G>A	0.50	90	0.48	373	0.56	348	0.38	146
3	Stop_Lost	*MYD88*	c.478T>C	0.29	86	0.37	249	0.50	199	0.35	121
22	Missense	*EP300*	c.4195G>A	0.25	53	0.29	163	0.49	82	0.32	53
6	Missense	*DST*	c.15124G>A	0.00	0	0.10	182	0.00	0	0.00	0
17	Missense	*CD79B*	c.632T>C	0.31	85	0.30	497	0.44	346	0.27	200
17	Missense	*CD79B*	c.599T>A	0.32	82	0.27	448	0.39	295	0.27	191
17	Splice_Region	*CD79B*	c.67+3A>G	0.34	120	0.31	678	0.44	516	0.26	261
X	Missense	*HEPH*	c.2284G>C	0.64	36	0.49	102	0.78	45	0.51	35
14	Frameshift	*ZFP36L1*	c.437delG	0.31	240	0.29	887	0.43	821	0.23	347
14	Missense	*ZFP36L1*	c.131G>A	0.41	143	0.26	283	0.42	204	0.18	105
12	synonymous	*BTG1*	c.108G>A	0.49	208	0.42	466	0.55	310	0.42	160
2	synonymous	*DUSP2*	c.429C>T	0.00	0	0.00	0	0.22	280	0.00	0
1	Missense	*CD58*	c.628G>A	0.31	74	0.36	247	0.68	142	0.38	80
11	Frameshift	*ETS1*	c.970_971 dupGT	0.28	134	0.29	476	0.27	531	0.31	190
11	Missense	*ETS1*	c.67C>T	0.25	88	0.28	634	0.51	667	0.22	134
3	Missense	*TBL1XR1*	c.1202C>A	0.30	50	0.24	168	0.40	100	0.36	42
3	Missense	*TBL1XR1*	c.1184A>G	0.24	41	0.24	153	0.40	86	0.38	39
2	synonymous	*UNC80*	c.8331G>A	0.38	53	0.21	222	0.35	185	0.21	67

Table S4. 7. Somatic, exonic mutations detected in patient 7.

Chr.	Variant Classification	Gene	cDNA Change	AF B1	Cov. B1	AF B2	Cov. B2	AF B3	Cov. B3	AF B4	Cov. B4
11	Missense	*DCHS1*	c.4588G>A	0.42	166	0.39	173	0.43	258	0.45	264
22	Splice_Donor	*IGLL5*	c.206+2T>G	0.31	145	0.37	145	0.36	168	0.42	151
6	Nonsense	*HLA-C*	c.573G>A	0.52	194	0.49	203	0.61	215	0.52	166
6	Splice_Donor	*PIM1*	c.189+1G>A	0.25	139	0.20	153	0.20	175	0.16	167
6	Missense	*PIM1*	c.550C>T	0.42	166	0.37	161	0.47	159	0.35	184
3	Stop_Lost	*MYD88*	c.478T>C	0.53	129	0.57	142	0.59	138	0.54	120
12	Nonsense	*KMT2D*	c.10201C>T	0.49	131	0.43	115	0.63	103	0.53	81
17	Missense	*CD79B*	c.676C>G	0.65	101	0.37	151	0.54	127	0.47	110
17	Missense	*CD79B*	c.589T>C	0.67	72	0.41	106	0.64	74	0.43	87
14	synonymous	*IGHJ5*	c.45C>T	0.44	18	0.46	24	0.57	23	0.48	42
2	synonymous	*LRP1B*	c.645G>A	0.33	18	0.36	33	0.25	16	0.36	11
6	Missense	*FNDC1*	c.1007G>A	0.24	98	0.13	129	0.18	83	0.13	67
3	Missense	*TBL1XR1*	c.683T>A	0.46	112	0.57	148	0.51	80	0.56	62
4	Missense	*TENM3*	c.2152G>A	0.16	255	0.12	322	0.14	195	0.13	226

Table S4. 8. Somatic, exonic mutations detected in patient 8.

Chr.	Variant Classification	Gene	cDNA Change	AF B1	Cov. B1	AF B2	Cov. B2	AF B3	Cov. B3	AF B4	Cov. B4
1	Inframe_Del	*TP73*	c.567_569 delCGT	0.37	221	0.26	179	0.18	216	0.24	281
17	Missense	*TP53*	c.537T>A	0.53	119	0.27	140	0.32	196	0.35	186
17	Missense	*TP53*	c.376T>C	0.64	64	0.37	73	0.25	118	0.41	92
19	Missense	*MUC16*	c.43304G>A	0.13	40	0.00	0	0.00	0	0.00	0
22	Missense	*IGLL5*	c.25_26del GGinsAA	0.20	74	0.12	76.5	0.11	107	0.11	94
22	synonymous	*IGLL5*	c.82C>T	0.39	83	0.29	91	0.31	107	0.29	83
22	Missense	*IGLL5*	c.128T>C	0.24	89	0.13	80	0.13	106	0.14	97
22	Missense	*IGLL5*	c.163T>C	0.39	87	0.26	80	0.32	123	0.27	104
22	Missense	*IGLL5*	c.206G>A	0.36	75	0.28	61	0.30	96	0.29	85
6	Missense	*HIST1H2AG*	c.279G>C	0.33	155	0.27	142	0.24	225	0.25	209
6	Missense	*HLA-B*	c.439T>G	0.14	154	0.12	112	0.08	127	0.09	136

8	Splice_Region	*TOX*	c.102+7G>A	0.00	0	0.08	100	0.12	183	0.06	127
17	Missense	*SDK2*	c.4547C>T	0.39	99	0.20	86	0.26	128	0.41	75
7	Nonsense	*SYPL1*	c.192T>A	0.41	46	0.25	71	0.30	109	0.30	76
12	Nonsense	*DTX1*	c.90G>A	0.32	145	0.10	114	0.19	149	0.27	132
12	synonymous	*BCL7A*	c.54C>A	0.16	68	0.00	0	0.00	0	0.00	0
5	synonymous	*PCDHB6*	c.879C>T	0.00	0	0.00	0	0.11	151	0.00	0
2	Missense	*LRP1B*	c.11816C>G	0.40	35	0.30	37	0.28	64	0.28	39
5	Missense	*FAT2*	c.9164C>T	0.27	55	0.26	53	0.19	96	0.28	64
2	Frameshift	*TTN*	c.58750delG	0.20	64	0.19	62	0.20	89	0.26	70
2	Missense	*TTN*	c.32086C>A	0.11	79	0.00	0	0.00	0	0.00	0

Table S4. 9. Somatic, exonic mutations detected in patient 9.

Chr.	Variant Classification	Gene	cDNA Change	AF B1	Cov. B1	AF B2	Cov. B2	AF B3	Cov. B3	AF B4	Cov. B4
6	synonymous	*IRF4*	c.60C>T	0.46	150	0.31	208	0.00	0	0.36	228
7	Missense	*CARD11*	c.1159G>C	0.12	118	0.00	0	0.00	0	0.00	0
12	Splice_Donor	*ETV6*	c.33+1G>A	0.68	65	0.47	150	0.42	146	0.47	140
22	synonymous	*IGLL5*	c.45G>A	0.35	97	0.34	168	0.63	174	0.41	205
22	Missense	*IGLL5*	c.70T>G	0.34	109	0.37	174	0.35	186	0.38	213
22	Missense	*IGLL5*	c.173G>A	0.44	114	0.32	173	0.26	178	0.36	192
22	synonymous	*IGLL5*	c.177C>T	0.43	114	0.30	172	0.25	179	0.35	180
22	Missense	*IGLL5*	c.182C>G	0.32	113	0.00	0	0.00	0	0.00	0
22	Splice_Region	*IGLL5*	c.206+4A>G	0.44	97	0.39	137	0.31	167	0.38	150
22	synonymous	*IGLL5*	c.261G>A	0.34	87	0.29	160	0.30	280	0.35	196
6	synonymous	*HIST1H2AC*	c.129G>A	0.39	241	0.28	323	0.32	394	0.34	394
6	Missense	*HIST1H1E*	c.616C>T	0.00	0	0.00	0	0.28	144	0.00	0
6	Initiator_Codon	*PIM1*	c.3G>A	0.35	252	0.24	357	0.27	292	0.34	422
6	synonymous	*PIM1*	c.21C>T	0.35	275	0.25	388	0.27	306	0.32	440
6	Missense	*PIM1*	c.41C>A	0.40	248	0.32	375	0.32	268	0.35	404
6	Missense	*PIM1*	c.72G>C	0.35	197	0.28	329	0.24	225	0.31	327
6	synonymous	*PIM1*	c.73C>T	0.00	0	0.00	0	0.00	0	0.11	320
6	Splice_Region	*PIM1*	c.82+8C>T	0.36	154	0.27	262	0.34	193	0.38	247
6	synonymous	*PIM1*	c.144C>T	0.00	0	0.00	0	0.13	111	0.00	0
6	Missense	*PIM1*	c.149G>A	0.51	89	0.43	143	0.33	115	0.48	139
6	Missense	*PIM1*	c.241C>G	0.28	74	0.21	87	0.00	0	0.24	134
6	Missense	*PIM1*	c.248G>A	0.30	83	0.20	93	0.08	185	0.25	150
6	Missense	*PIM1*	c.264G>T	0.00	0	0.21	87	0.00	0	0.00	0
6	synonymous	*PIM1*	c.277C>T	0.38	92	0.21	86	0.43	172	0.33	178
6	Missense	*PIM1*	c.286G>A	0.39	97	0.24	90	0.42	173	0.32	195
6	synonymous	*PIM1*	c.300C>T	0.00	0	0.13	101	0.00	0	0.00	0
6	Nonsense	*PIM1*	c.327G>A	0.37	98	0.25	123	0.00	0	0.37	213
6	Nonsense	*PIM1*	c.379C>T	0.00	0	0.00	0	0.00	0	0.21	231
6	Nonsense	*PIM1*	c.403G>T	0.17	110	0.00	0	0.00	0	0.00	0
6	Frameshift	*PIM1*	c.415_437del CTGCAAGAG GAGCTGGCC CGCAG	0.00	0	0.00	0	0.21	263	0.00	0
6	Missense	*PIM1*	c.490C>T	0.00	0	0.28	185	0.00	0	0.00	0
6	synonymous	*PIM1*	c.492C>G	0.39	104	0.35	183	0.20	238	0.33	188
6	synonymous	*PIM1*	c.495C>T	0.00	0	0.00	0	0.21	240	0.00	0
6	synonymous	*PIM1*	c.543G>A	0.00	0	0.00	0	0.31	243	0.00	0
6	Missense	*PIM1*	c.549G>C	0.38	116	0.31	213	0.29	235	0.35	207
6	Missense	*PIM1*	c.550C>T	0.43	116	0.38	212	0.33	231	0.38	208
6	synonymous	*PIM1*	c.579C>T	0.34	112	0.31	185	0.31	195	0.34	191
6	Missense	*PIM1*	c.673G>A	0.45	141	0.33	222	0.31	244	0.34	267
6	Nonsense	*PIM1*	c.867G>A	0.45	100	0.31	214	0.00	0	0.39	241

12	Frameshift	*KMT2D*	c.8376_8379delAGGA	0.57	116	0.50	144	0.42	239	0.52	182
6	Missense	*DST*	c.5744T>A	0.31	81	0.37	107	0.29	153	0.26	137
8	Missense	*TOX*	c.953A>G	0.79	105	0.66	130	0.29	136	0.63	156
8	Splice_Acceptor	*TOX*	c.925-2A>C	0.79	91	0.65	101	0.28	117	0.60	126
18	synonymous	*BCL2*	c.24G>A	0.15	298	0.18	528	0.17	630	0.18	615
17	Missense	*CD79B*	c.589T>C	0.75	51	0.68	68	0.60	135	0.70	81
17	Missense	*RP11-1055B8.7*	c.4541T>C	0.82	204	0.59	258	0.58	217	0.72	304
12	Splice_Region	*BTG1*	c.148+5G>C	0.30	198	0.25	290	0.26	251	0.25	280
12	synonymous	*BTG1*	c.78C>G	0.00	0	0.00	0	0.00	0	0.12	334
8	Splice_Region	*MYC*	c.-153C>T	0.77	75	0.69	108	0.34	175	0.76	143
8	Missense	*MYC*	c.475C>T	0.76	250	0.69	352	0.35	376	0.72	411
2	Missense	*LRP1B*	c.1309G>A	0.00	0	0.00	0	0.28	60	0.00	0
2	Missense	*TTN*	c.10598A>C	0.41	74	0.29	130	0.28	166	0.34	176
3	Missense	*KLHL6*	c.247T>A	0.43	76	0.32	151	0.30	178	0.36	191
3	Missense	*KLHL6*	c.185T>C	0.40	75	0.32	189	0.29	216	0.39	247
4	Missense	*TENM3*	c.7080G>T	0.43	84	0.38	109	0.30	156	0.27	176

Table S4. 10. Somatic, exonic mutations detected in patient 10.

Chr.	Variant Classification	Gene	cDNA Change	AF B1	Cov. B1	AF B2	Cov. B2	AF B3	Cov. B3	AF B4	Cov. B4	AF B5	Cov. B5
1	Frameshift	*TNFRSF14*	c.160delT	0.76	162	0.79	163	0.83	134	0.82	165	0.92	158
17	Missense	*TP53*	c.613T>G	0.78	303	0.71	299	0.80	219	0.81	278	0.88	202
19	Missense	*MUC16*	c.27509C>T	0.42	530	0.40	543	0.43	387	0.47	348	0.41	241
19	Missense	*MUC16*	c.23767G>A	0.00	0	0.00	0	0.00	0	0.00	0	0.22	230
X	Splice_Acceptor	*TMSB4X*	c.-16-1G>A	0.00	0	0.00	0	0.10	305	0.00	0	0.00	0
X	Missense	*TMSB4X*	c.117G>C	0.42	266	0.37	278	0.44	220	0.26	129	0.41	142
6	Splice_Acceptor	*CD83*	c.38-1G>A	0.39	366	0.36	338	0.00	0	0.00	0	0.00	0
6	Missense	*CD83*	c.80G>A	0.40	438	0.38	0	0.00	0	0.00	0	0.00	0
6	Missense	*CD83*	c.130C>T	0.00	0	0.00	0	0.00	0	0.19	497	0.00	0
22	Missense	*IGLL5*	c.116T>G	0.48	145	0.63	161	0.81	85	0.64	162	0.59	164
22	Missense	*IGLL5*	c.137A>C	0.36	203	0.31	190	0.00	0	0.35	201	0.41	205
22	Missense	*IGLL5*	c.166G>A	0.39	203	0.40	178	0.75	73	0.45	170	0.38	196
22	Missense	*IGLL5*	c.182C>G	0.33	178	0.35	162	0.77	57	0.41	153	0.32	179
22	synonymous	*IGLL5*	c.195C>T	0.31	156	0.31	149	0.74	46	0.38	134	0.28	152
6	Missense	*HIST1H1E*	c.541C>T	0.44	273	0.36	197	0.44	200	0.42	257	0.53	220
6	synonymous	*HIST1H1D*	c.186G>A	0.43	474	0.37	550	0.45	468	0.42	569	0.42	511
6	Splice_Region	*PIM1*	c.82+7G>C	0.28	443	0.28	352	0.31	297	0.33	454	0.32	500
6	Missense	*PIM1*	c.248G>C	0.00	0	0.00	0	0.00	0	0.00	0	0.22	560
6	synonymous	*PIM1*	c.549G>A	0.00	0	0.00	0	0.00	0	0.14	451	0.31	429
13	Missense	*POSTN*	c.2249G>A	0.37	132	0.26	107	0.51	140	0.32	62	0.31	187
12	Nonsense	*KMT2D*	c.7900C>T	0.47	392	0.39	358	0.41	238	0.44	368	0.40	375
6	Nonsense	*DST*	c.3238C>T	0.44	156	0.40	121	0.44	170	0.33	98	0.46	139
6	synonymous	*DST*	c.1101C>A	0.39	187	0.37	134	0.42	170	0.44	96	0.37	121
X	Missense	*TAF1*	c.91G>C	0.39	264	0.38	271	0.40	219	0.31	216	0.48	279
8	Missense	*ZFHX4*	c.6760G>A	0.48	318	0.38	274	0.50	296	0.34	217	0.44	227
7	Missense	*PCLO*	c.7381A>G	0.22	505	0.20	508	0.28	293	0.32	270	0.33	283
7	Missense	*PCLO*	c.2436A>C	0.48	852	0.44	848	0.00	0	0.00	0	0.00	0
16	Missense	*IRF8*	c.259A>T	0.88	528	0.84	641	0.92	419	0.89	377	0.91	266
16	Missense	*IRF8*	c.272A>C	0.87	559	0.84	669	0.92	435	0.88	387	0.91	280
10	Missense	*FAS*	c.324T>A	0.46	451	0.40	424	0.48	369	0.42	271	0.46	228
10	Frameshift	*FAS*	c.396_397insT	0.37	325	0.36	352	0.35	412	0.35	258	0.42	260
2	synonymous	*DUSP2*	c.451C>T	0.11	150	0.00	0	0.00	0	0.00	0	0.00	0
2	Missense	*DUSP2*	c.428G>A	0.41	154	0.38	166	0.43	103	0.39	118	0.43	141
2	Splice_Region	*DUSP2*	c.388+5G>C	0.00	0	0.00	0	0.00	0	0.00	0	0.38	79

Table S4. 11. Somatic, exonic mutations detected in patient 11.

Chr.	Variant Classification	Gene	cDNA Change	AF B1	Cov. B1	AF B2	Cov. B2	AF B3	Cov. B3	AF B4	Cov. B4	AF B5	Cov. B5
17	Missense	*TP53*	c.752T>G	0.13	271	0.21	126	0.12	136	0.11	243	0.10	187
9	Nonsense	*CDKN2A*	c.238C>T	0.00	0	0.17	117	0.00	0	0.00	0	0.00	0
14	Nonsense	*NFKBIA*	c.126C>G	0.06	309	0.12	139	0.05	191	0.00	0	0.00	0
22	Missense	*EP300*	c.4336T>C	0.11	327	0.21	478	0.07	203	0.09	289	0.08	158
12	Nonsense	*KMT2D*	c.5674C>T	0.17	292	0.26	185	0.18	126	0.17	179	0.22	160
4	synonymous	*WDFY3*	c.9987C>T	0.12	389	0.20	191	0.12	215	0.07	391	0.10	365

Table S4. 12. Somatic, exonic mutations detected in patient 12.

Chr.	Variant Classification	Gene	cDNA Change	AF B1	Cov. B1	AF B2	Cov. B2	AF B3	Cov. B3	AF B4	Cov. B4
X	Missense	*P2RY8*	c.508C>G	0.00	0	0.00	0	0.18	375	0.00	0
X	Missense	*P2RY8*	c.229C>A	0.11	335	0.81	335	0.41	406	0.76	309
X	Missense	*P2RY8*	c.203C>T	0.55	330	0.00	0	0.00	0	0.00	0
7	Missense	*CARD11*	c.644A>C	0.39	296	0.41	352	0.46	344	0.46	259
22	Missense	*IGLL5*	c.45G>C	0.39	174	0.51	278	0.45	316	0.46	219
22	Missense	*IGLL5*	c.85C>G	0.41	196	0.47	304	0.44	343	0.44	238
22	Inframe_Del	*IGLL5*	c.172_174delAGC	0.37	213	0.42	325	0.43	363	0.43	221
22	Splice_Region	*IGLL5*	c.206+3A>C	0.40	130	0.48	220	0.45	238	0.44	169
6	Missense	*PIM1*	c.244A>G	0.32	210	0.00	0	0.00	0	0.00	0
13	Initiator_Codon	*FOXO1*	c.1A>G	0.41	22	0.52	29	0.40	50	0.44	34
13	Missense	*RB1*	c.44C>T	0.43	141	0.59	183	0.59	269	0.60	165
14	Missense	*NRXN3*	c.304T>G	0.00	0	0.00	0	0.12	193	0.00	0
3	Missense	*ABI3BP*	c.3016A>C	0.00	0	0.00	0	0.00	0	0.13	150
8	Missense	*CSMD3*	c.9110T>C	0.00	0	0.46	117	0.25	125	0.47	110
8	Splice_Region	*CSMD3*	c.4896-3C>T	0.00	0	0.00	0	0.00	0	0.45	60

Table S5. Suggested driver mutations with stable allelic frequencies (AF) across all biopsies (B) of a patient.

Patient	Gene	cDNA Change	Median Tumour Cell Content (Range)	AF B1	AF B2	AF B3	AF B4	AF B5	AF B6	AF B7
1	*MYD88*	c.478T>C	80% (40–90%)	0.43	0.37	0.41	0.46	0.45	0.42	n.a.
2	*NOTCH2*	c.6909delC	90% (70–90%)	0.38	0.42	0.37	0.53	0.39	n.a.	n.a.
3	*MYD88*	c.719T>C	90% (80–90%)	0.42	0.58	0.56	0.36	n.a.	n.a.	n.a.
4	*TP53*	c.526T>C	90% (80–90%)	0.32	0.44	0.38	0.38	0.40	n.a.	n.a.
5	*TP53*	c.517G>A	90% (80–90%)	0.81	0.92	0.85	0.72	0.82	0.70	0.53
6	*TP53*	c.376-1G>A	75% (70–90%)	0.40	0.47	0.75	0.47	n.a.	n.a.	n.a.
7	*MYD88*	c.478T>C	80% (80–90%)	0.53	0.57	0.59	0.54	n.a.	n.a.	n.a.
8	*TP53*	c.537T>A	60% (50–70%)	0.53	0.27	0.32	0.35	n.a.	n.a.	n.a.
9	*KMT2D*	c.8376_8379delAGGA	85% (80–90%)	0.57	0.50	0.42	0.52	n.a.	n.a.	n.a.
10	*TP53*	c.613T>G	90% (90%)	0.78	0.71	0.80	0.81	0.88	n.a.	n.a.
11	*TP53*	c.752T>G	80% (70–80%)	0.13	0.21	0.12	0.11	0.10	n.a.	n.a.
12	*CARD11*	c.644A>C	90% (90%)	0.39	0.41	0.46	0.46	n.a.	n.a.	n.a.

AF = allelic frequency, B = biopsy, n.a. = not available.

Table S6. Selected genes for exon sequencing.

Selected Genes					
ABCC8	***CDKN2A***	***FAT4***	***KMT2D***	***P2RY8***	*STAT3*
ABI3BP	*CDKN2B*	*FBLN2*	*KRAS*	*PASD1*	*SUSD2*
ACTB	*CIITA*	*FGFRL1*	*KRTAP5-5*	*PASK*	*SYPL1*
ACTG1	*CNTNAP5*	*FNDC1*	*LRP1B*	*PBMUCL1=MUC22*	*TAF1*
ADAMTS5	*COL12A1*	*FOXO1*	*LRRN3*	*PCDH7*	*TBC1D4*
ADAMTSL3	*CPEB2*	*GNA13*	*LYN*	*PCDHB11*	*TBL1XR1*

AKAP8	*CPS1*	*GNAI2*	*MAGEC3*	*PCDHB6*	*TCHH*
ALDH3A2	*CREBBP*	*GPR37*	*MAP2K1*	*PCLO*	*THBS4*
ANKLE2	*CSMD3*	*HDAC7*	*MCL1*	*PDS5B*	*TLL2*
APC2	*CXCR4*	*HEATR7B2*	*MED12L*	*PIM1*	*TMEM30A*
ATM	*DCDC5*	*HEPH*	*MEF2B*	*PKD1*	*TMSB4X*
B2M	*DCHS1*	*HERC2*	*MEF2BNB*	*PMS1*	*TMSL3*
BAHCC1	*DDX3X*	*HIST1H1C*	*MEP1B*	*POGZ*	*TNFAIP*
BAI1	*DIAPH2*	*HIST1H1D*	*MIF4GD*	*POSTN*	*TNFAIP3*
BCL10	*DNAH5*	*HIST1H1E*	*MPDZ*	*POU2AF1*	*TNFRSF14*
BCL2	*DNAH7*	*HIST1H2AC*	*MPEG1*	*POU2F2*	*TOX*
BCL2s	*DPYD*	*HIST1H2AG*	*MTMR8*	*PRDM1*	*TP53*
BCL6	*DPYS*	*HIST1H3B*	*MUC16*	*PRKCB*	*TP73*
BCL7A	*DSC3*	*HLA-A*	*MYC*	*PRKCD*	*TRAF3*
BCR	*DSEL*	*HLA-B*	*MYD88*	*PTEN*	*TRIM2*
BRAF	*DSG4*	*HMCN1*	*MYH4*	*PTPN23*	*TSC22D1*
BTG1	*DST*	*HNF1B*	*MYO19*	*RB1*	*TTN*
BTG2	*DTX1*	*IER2*	*MYOM2*	*ROBO2*	*UBE2A*
C10orf12	*DUSP2*	*IFNGR1*	*NFKBIA*	*S1PR2*	*UNC5C*
C10orf71	*DUSP27*	*IGLL5*	*NLRP5*	*SALL3*	*UNC5D*
C12orf35	*EBF1*	*IKZF3*	*NOTCH1*	*SAMD9L*	*UNC80*
CARD11	*EEF1A1*	*IRF2BP2*	*NOTCH2*	*SARM1*	*WDFY3*
CCND3	*EIF4A2*	*IRF4*	*NRXN3*	*SDK2*	*ZAN*
CD36	*EP300*	*IRF8*	*OBSCN*	*SEC14L5*	*ZFHX4*
CD37	*ETS1*	*ITPKB*	*ODZ2*	*SGK1*	*ZFP36L1*
CD58	*ETV6*	*KDM2B*	*ODZ3*	*SLC2A12*	*ZNF471*
CD70	*EZH2*	*KIAA1614*	*ODZ4=TENM4*	*SLITRK3*	*ZNF608*
CD79B	*FAM38A*	*KIF1C*	*OFD1*	*SLITRK6*	*ZNF804A*
CD83	*FAM38B*	*KLF2*	*OR10A2*	*SMARCA4*	
CDH9	*FAS*	*KLHL14*	*OSBPL10*	*SOCS1*	
CDKN1B	*FAT2*	*KLHL6*	*P2RX5*	*SRRM2*	
